# Emerging feline vector-borne pathogens in Italy

**DOI:** 10.1186/s13071-019-3409-8

**Published:** 2019-05-02

**Authors:** Giulia Morganti, Fabrizia Veronesi, Valentina Stefanetti, Trentina Di Muccio, Eleonora Fiorentino, Manuela Diaferia, Azzurra Santoro, Fabrizio Passamonti, Marina Gramiccia

**Affiliations:** 1 Department of Veterinary Medicine, University of Perugia, Via San Costanzo 4, 06126 Perugia, Italy; 2 Department of Infectious Diseases, Unit of Vector-borne Diseases, Istituto Superiore di Sanità, Viale Regina Elena 299, 00161 Rome, Italy

**Keywords:** Cat, *Babesia* spp., *Cytauxzoon* spp., *Leishmania infantum*, *Rickettsia* spp.

## Abstract

**Background:**

The epidemiology of feline vector-borne pathogens (FeVBPs) has been less investigated in cats than in dogs. The present study assessed the prevalence of *Rickettsia* spp., *Babesia* spp., *Cytauxzoon* spp. and *Leishmania infantum* infections in cat populations living in central Italy, by molecular and serological tools.

**Results:**

A total of 286 healthy cats were randomly selected from catteries and colonies in central Italy. Peripheral blood and conjunctival swab (CS) samples were collected during surgical procedures for regional neutering projects. Sera were analysed by IFAT to detect anti-*Rickettsia felis*, *R. conorii*, *Babesia microti* and *Leishmania* IgG antibodies using commercial and home-made antigens. DNA extracted from buffy coats (BCs) was tested for *Rickettsia* spp., and Piroplasmida species, including *Cytauxzoon* spp. and *Babesia* spp. by PCR. Buffy coats and CS samples were assayed by a nested (n)-PCR for *Leishmania* spp. Sixty-two cats (21.67%) were seropositive to at least one of the tested pathogens. The serological assay revealed 23 (8.04%) and 18 (6.29%) positive cats for *R. felis* and *R. conorii*, respectively, with low titers (1/64–1/128). No antibodies against *B. microti* were detected. Neither *Rickettsia* nor Piroplasmida DNA were amplified using the specific PCR assays. Thirty-one cats (10.83%) tested positive to anti-*Leishmania* IgG, with titers ranging from 1:40 to 1:160 and 45 animals (15.73%) tested positive to *Leishmania* CS n-PCR, whereas none of the animals tested positive to BC n-PCR. Considering the results obtained by IFAT and CS n-PCR, a moderate agreement between the two tests was detected (κ = 0.27).

**Conclusions:**

The results of the serological and molecular surveys showed a moderate exposure to *Leishmania* in the investigated cats and highlighted the limited molecular diagnostic value of BC *versus* CS samples for this pathogen. Conversely no evidence supported the circulation of *Cytauxzoon* spp. in domestic cats, in contrast with previous detections in European wild cats in the same areas monitored. The low positive titres for *R. felis* in association with no DNA BC amplification prevent speculation on the exposure of feline populations to this FeVBP due to the cross-reactivity existing within spotted fever group rickettsiosis (SFGR).

## Background

Cats, especially those with an outdoor lifestyle, are highly likely to be exposed to several arthropods such as fleas, ticks and sand flies, and consequently to the pathogens that they potentially harbor [1]. Although cats may act as carriers of infected arthropods to humans and other pets that share the domestic habitat, in Europe the epidemiology of feline vector-borne pathogens (FeVBPs) is generally less investigated in cats than in dogs [2]. Other than bartonellosis by *Bartonella henselae* and feline infectious anemia by *Mycoplasma haemofelis*, for which a large amount of literature has been generated in the last decade [3, 4], there is evidence that cat populations are exposed to several other emerging VBPs, including those of zoonotic concern, although they have been scarcely investigated [1, 5]. Species belonging to the *Rickettsiaceae* family, piroplasmids and *Leishmania infantum* are some of the FeVBPs that are worthy of investigation in order to clarify the potential role of domestic cats in maintaining and distributing these microorganisms to humans and other animal species, e.g. dogs in endemic areas.

In fact, cats may play a role as sentinels for some Rickettsiae of the spotted fever group (SFG), e.g. *Rickettsia conorii* and *Rickettsia felis* [6, 7]. *Rickettsia* *conorii* is historically the most important zoonotic SFG species in the Mediterranean area [8] which is transmitted by ticks. The role of the dog as a reservoir and epidemiological sentinel for *R. conorii* infection is well documented [9, 10]; however, some serological surveys have recently shown that cats exposed to *Riphicephalus sanguineus*-bites develop a detectable antibody response against *R. conorii*, reaching up to 44% seroprevalence in some European countries [7, 8, 11]. Seroreactivity against *R. conorii *has been observed in cats from the islands and southern regions of Italy with prevalence rates ranging from 7 to 48.7% [12]. *Rickettsia felis* is the agent of the emerging flea-borne spotted fever (FBSV), also known as cat flea typhus, whose human clinical cases have been reported worldwide [13, 14]. Cats experimentally and naturally exposed to *R. felis* infected fleas of the species *Ctenocephalides felis* were found to become seropositive [15] and the pathogen was detectable by PCR in naïve fleas feeding on infected cats, as expression of an active bacteremia [16].

Feline piroplasmoses are tick-borne infections caused by agents of the genera *Cytauxzoon* and *Babesia*. *Cytauxzoon* infections have been largely documented in wild and domestic felids in Europe in the last few years [17–23]. The Iberian lynx (*Lynx pardinus*) and the European wild cat (*Felis silvestris silvestris*) have been found to be infected with the *Cytauxzoon* spp., genetically and pathogenetically different from the American species *Cytauxzoon felis* [22, 24]. This thus suggests their reservoir role in the sylvatic cycle and also the possibility of transmission to domestic cats where the habitat is shared with wildlife. In Italy, evidence of the presence of *Cytauxzoon* spp. has been provided both in wild [22] and domestic cats (in north-eastern and central Italy), also in association with sporadic cases of disease [17, 23]. In any case, such infections are likely to be underdiagnosed and causes and impacts of feline cytauxzoonosis have not yet been clarified. On the other hand, reports on the presence of other piroplasmids, primarily associated with dogs, such as *Babesia vogeli* and *Babesia canis* have been described very sporadically in cats from Europe [25, 26]. In Italy, *Babesia microti* DNA has been detected in the blood samples of cats [27] and also a high rate of seroreactivity (20.3–23.8%) has been observed [5, 12], indicating the possible role of cats as the host and epidemiological sentinel for human babesiosis agents.

Zoonotic visceral leishmaniasis (ZVL) caused by the protozoan *Leishmania infantum*, is a sand fly-borne human disease which is endemic in the Mediterranean Basin, Middle East, Asia, and Central and South America [28]. The domestic dog is the principal reservoir host and plays a central role in *L. infantum* transmission to humans. Canine leishmaniosis (CanL) is a severe disease which is difficult to manage since both symptomatic and asymptomatic dogs can be infectious for phlebotomine vectors. The role of other mammalian hosts in the parasite life-cycle has also been investigated and cannot be ruled out [29]. Sero-epidemiological and molecular surveys have been performed on cats in Mediterranean countries [30]. In Italy, several studies have detected antibodies and/or *L. infantum* DNA in cat populations, with a wide prevalence range, showing how this animal is exposed to *Leishmania* infection [1, 5, 12, 31, 32]. Furthermore, experimental evidence by xenodiagnosis has proved that naturally infected cats are infectious to phlebotomine vectors [33]. These results suggest that cats act as ZVL reservoir hosts and could be included in the range of secondary hosts in CanL endemic areas. Feline leishmaniosis (FeL) can be considered as an emergent FeVBP not only from an epidemiological but also a clinical perspective [30, 34]. In fact, an increasing number of cats exhibit clinical symptoms which in most cases appear less severe than in CanL but are, however, difficult to treat and prevent [8, 30, 34].

The present study assesses the prevalence of *Rickettsia* spp., *Cytauxzoon* spp., *Babesia* spp. and *L. infantum* infections by molecular and serological techniques in cat populations living in Italy in areas that have received little investigation and which are endemic for canine VBPs.

## Methods

### Animal sampling

The study was carried out from 2010 to 2016 on a total sample of 286 cats from catteries and colonies of central Italy (Umbria, Tuscany and Marche regions), an area that is endemic for canine VBPs [35–37]. The animals were recruited during surgical procedures related to regional neutering projects, following written content from the person in charge of the cat shelter or colony of origin. Cats were submitted before surgery to a physical examination for signs associated with FeL, i.e. skin and ocular lesions and to unspecific clinical signs such as fever, apathy, anorexia, weight loss, pallor, lymphadenopathy, splenomegaly, gastrointestinal alterations and gingivostomatitis, in accordance with the list provided by Persichetti et al. [12]. Information on ectoparasiticide treatment and presence of ectoparasites was also collected.

Peripheral blood (2.5 ml) was obtained from the jugular vein and equally distributed into ethylenediamine tetraacetic acid (EDTA)-coated and without-EDTA tubes. Sera and buffy coats (BCs) were obtained by centrifugation of blood samples at 2500× *g* for 10 min and frozen at -20 °C until further use for serological and biomolecular investigations. Exfoliative epithelial cells were also collected from the right and left conjunctiva of each animal using sterile cotton swabs manufactured for bacteriological isolation. The conjunctival swabs (CSs) were rubbed vigorously back and forth in the lower conjunctival sac, and then immersed in 2 ml of sterile saline in 20-ml plastic tubes. After manual stirring of the swabs, the saline containing the eluted exfoliating cells was centrifuged at 6000× *g* for 10 min, the supernatant was eliminated and the cells were stored at -20 °C pending DNA extraction [37]. Samples from the right and left eyelid conjunctivas were processed together.

### Serological analysis

The presence of immunoglobulin G (IgG) against *R. conorii*, *R. felis* and *B. microti* antigens was assessed by indirect fluorescent antibody test (IFAT) using commercial antigens, i.e. slides coated with purified individual substrate antigens of *R. felis* (Fuller Laboratories, Fullerton, CA, USA), slides coated with *R. conorii* antigens (Mega Cor Diagnostik GmbH, Hörbranz, Austria) and slides coated with merozoites of *B. microti* (MegaFLUO^®^ *Babesia microti*, Mega Cor Diagnostik GmbH).

For the detection of anti-*Leishmania* IgG, sera were tested with a homemade IFAT following the standard procedures recommended by the Office International des Epizooties [38] and using promastigotes of *L. infantum* zymodeme MON-1 (MHOM/TN/80/IPT-1) as a source of antigen.

For all the serological tests, commercial anti-feline IgG polyclonal antiserum conjugated to fluorescein isothiocyanate (MegaFluo^®^ FITC IgG, MegaCor Diagnostik GmbH; working dilution 1/100) was used as conjugate. Positive and negative controls provided by the commercial kits were added to each specific reaction for *R. conorii*, *R. felis* and *B. microti*; however, positive and negative controls for *Leishmania* consisted on sera obtained from a cytologically-confirmed clinical illness cat, and from a cat previously tested negative by serological and molecular assays, respectively.

The results obtained were interpreted using the cut-off dilutions of 1/64 for *R. conorii*, *R. felis* and *B. microti* [7, 12] and 1/40 for *L. infantum* [33, 39]. End-point titers of the positive serum samples were determined for all the FeVBPs investigated.

*Cytauxzoon* spp. was not serologically investigated due to the lack of commercially available antigens.

### Biomolecular techniques

Total DNA was extracted from BCs using the QIAamp^®^ DNA Blood Mini Kit (Qiagen GmbH, Hilden, Germany), following the manufacturer’s protocol. For the DNA extraction of the samples containing eluted conjunctival cells, a traditional method was performed and the obtained pellets were resuspended in 90 μl of lysis buffer plus 10 μl of 2 mg/ml proteinase K. After 2 h of incubation at 56 °C, the proteinase K was inactivated at 95 °C for 10 min; the samples were then centrifuged at 13,000× g for 10 min. Supernatants were collected in 1.5-ml vials and stored at -20 °C pending PCR assays.

Each sample of BC-DNA was used as a template in a nested-(n)PCR assay for *Rickettsia* spp., targeting fragments of the *gltA* [40], *ompB* [41] and *ompA* [42] rickettsial genes. A conventional PCR reaction, which amplified a portion of the small subunit ribosomal DNA (*SSU* rDNA) of the Piroplasmida species was performed [43].

Total genomic DNA from both BC and CS was also tested by *SSU* rDNA n-PCR assay for the presence of *Leishmania*. The n-PCR protocol was selected due its well-standardized diagnostic procedure and well-known high sensitivity and specificity [37, 44] in endemic *L. infantum* CanL foci in Italy [44].

The primer sets and the thermal conditions used in the PCR assays performed are reported in Table 1. Negative (sterile water) and positive controls were included in each run. Positive controls consisted of DNA extracted from sequenced diagnostic samples that were positive for *Rickettsia monacensis*, *Cytauxzoon* spp., and *L. infantum*.

**Table 1:** Pathogens, target genes, nucleotide sequences, primer melting temperatures, amplicon sizes (in base pairs) and references

Target species	Target gene	Primer sequence (5′–3′)	Tm (°C)	Amplicon size (bp)	Reference
*Rickettsia* spp.	*gltA*	GGGGGCCTGCTCACGGCGG	54	381	[40]
		ATTGCAAAAAGTACAGTGAACA			
	*ompB*	GTAACCGGAAGTAATCGTTTCGTAA	54	511	[41]
		GCTTTATAACCAGCTAAACCACC			
		GTTTAATACGTGCTGCTAACCAA	56	425	
		GGTTTGGCCCATATACCATAAG			
	*ompA*	ATGGCGAATATTTCTCCAAAA	50	532	[42]
		AGTGCAGCATTCGCTCCCCCT			
Piroplasmida	*18S* rRNA	AATACCCAATCCTGACACAGGG	57	408	[43]
		TTAAATACGAATGCCCCCAAC			
*Leishmania* spp.	*18S* rRNA	GGTTCCTTTCCTGATTTACG	60	603	[37]
		GGCCGGTAAAGGCCGAATAG			
		TCCCATCGCAACCTCGGTT		358	[44]
		AAAGCGGGCGCGGTGCTG			

### Statistical analysis

The cross-sectional prevalence of infection was calculated as the rate of animals that were positive to each test for each pathogen checked. McNemar’s test for symmetry was used to compare the positive results between CS n-PCR and IFAT, and the agreement between tests was assessed using Cohen’s kappa coefficient (κ); a κ value of 0.21 to 0.60 represents a fair to moderate agreement, a κ value of 0.60 to 0.80 represents substantial agreement, and a κ value > 0.80 represents almost perfect agreement. Possible associations between infections and categorical variables including sex, geographical origin, age, living arrangement, and ectoparasitic status were assessed using Pearson’s Chi-square and Fisher’s exact test, as appropriate.

## Results

One hundred sixty-six out of the total of 286 cats were collected from the Province of Perugia (Umbria region), 22 from Macerata (Marche region) and 98 from Arezzo (Tuscany region). The animals were of both sexes (162 females and 124 males) and 254 animals were aged ≥ 1 year and 32 cats were aged < 1 year. A total of 112 animals came from colonies and 174 belonged to catteries. The cats were in good physical condition, and did not show apparent clinical signs and no historical evidence of recent anti-parasitic drug treatments was referred.

Cats were visually inspected for flea and tick infestations. In detail, 164 animals (57.34%, 95% CI: 51.61–63.07%) had flea infestation, 23 (8.04%, 95% CI: 4.89–11.19%) had ticks while 20 (6.99%, 95% CI: 4.04–9.95%) were co-infested. Although detected in 167 out of 286 individuals (58.39%, 95% CI: 52.68–64.10%), ectoparasites were not collected.

Sixty-two cats (21.67%) tested serologically positive to at least one of the tested pathogens, while 10 (3.5%) were seropositive to two pathogens; the prevalence of infection with different pathogens is summarized in Table 2.

**Table 2:** Prevalence of infections with the investigated FeVBPs determined by serology and PCR among the 286 cats

Pathogen	No. of seropositive cats (%) [95% CI]	No. of PCR-positive cats (%) [95% CI]
Single infections	52 (18.18) [13.71–22.65]	45 (15.73) [11.51–19.95]^a,b^
*Leishmania infantum*	26 (9.09) [5.76–12.42]	0 (0)^a^; 45 (15.73) [11.51–19.95]^b^
*Rickettsia conorii*	10 (3.49) [1.37–5.63]	0 (0)^a^
*Rickettsia felis*	16 (5.59) [2.93–8.26]	0 (0)^a^
*Cytauxzoon* spp.	nt	0 (0)^a^
*Babesia* spp.	0 (0)	0 (0)^a^
Co-infections	10 (3.5) [1.37–5.63]	0 (0)^a,b^
*Leishmania infantum* *+* *Rickettsia conorii*	3 (1.05) [0–2.23]	0 (0)^a^
*Leishmania infantum* *+* *Rickettsia felis*	2 (0.7) [0–1.67]	0 (0)^a^
*Rickettsia conorii* *+* *Rickettsia felis*	5 (1.75) [0.23–3.27]	0 (0)^a^
Total	62 (21.67) [16.9–26.45]	45 (15.73) [11.51–19.95]^a,b^

^a^PCR performed on buffy coat

^b^PCR performed on conjunctival swab

*Abbreviation*: nt, not tested

The serological assay for *R. felis* revealed 23 positives (8.04%, 95% CI: 4.89–11.19%) at a low titer (1/64). However, the prevalence of anti-*R. conorii *antibodies was 6.29% (18 animals) with titers ranging from 1/64 to 1/128. No antibodies against *B. microti* were detected. Thirty-one cats (10.83%, 95% CI: 7.24–14.44%) were positive for anti-*Leishmania* IgG, with titers ranging from 1:40 to 1:160. Specifically, 21 cats (7.34%) had a titer of 1:40, 6 (2.09%) of 1:80, and 4 (1.39%) of 1:160. Concurrent antibody positivity against *L. infantum* and *R. conorii* and or *R. felis* was found in 5 cats (1.75%, 95% CI: 0.23–3.27%) and against *R. felis* and *R. conorii* in 5 cats (1.75%, 95% CI: 0.23–3.27%).

No *Rickettsia* or piroplasmid DNA was amplified using the specific PCR assays; however, 45 animals (15.73%) tested positive to *Leishmania* CS n-PCR, whereas none of the BC samples were molecularly positive for *Leishmania* DNA.

Considering the results obtained by IFAT and CS n-PCR, the overall rate of *Leishmania* positives was 21.67% (62 out of 286 animals), of which 14 animals (4.89%) tested positive to both tests, 31 cats (10.83%) tested positive only to CS n-PCR, and 17 (5.94%) tested only IFAT-positive, with moderate agreement between the two tests (κ = 0.27).

The univariate analysis showed that several variables had a significant association with molecular and/or serological detection of FeVBPs. A significant association between exposure to *L. infantum* and geographical area of sampling was revealed both at serological (*χ*^2^ = 28.952, *df* = 2, *P* < 0.0001) and biomolecular (*χ*^2^ = 26.545, *df* = 2, *P* < 0.0001) levels. In particular, the animals from Tuscany showed a significantly higher rate of positivity than those sampled in Umbria and Marche. Moreover, adult cats had a significantly higher risk of being found serologically positive to *L. infantum* (*χ*^2^ = 4.38, *df* = 1, *P* = 0.036), *R. conorii* (*χ*^2^ = 4.64, *df* = 1, *P* = 0.042) and *R. felis* (*χ*^2^ = 4.82, *df* = 1, *P* = 0.046) than cats that were less than one year of age.

The housing arrangement significantly influenced the exposure rates to FeVBPs. In fact, cats from colonies showed a higher level of exposure to *L. infantum* than cats housed in catteries. On the other hand, animals from catteries showed a higher seropositivity both for *R. conorii* and *R. felis* (Table 3). There was a non-significant difference in FeVBPs prevalence rates between males and females, and a non-significant difference for animals infested or not infested by ectoparasites with the exception of *R. felis* seropositivity which was significantly higher in cats with a flea infestation (*χ*^2^ = 6.03, *df* = 1, *P* = 0.014).

**Table 3:** Comparison of prevalence of infectious agents detected by molecular and serological procedure in cats per categorical variables

Variable/category	No. of cats	*Leishmania infantum*			*Rickettsia conorii*		*Rickettsia felis*		*Cytauxzoon* spp.	*Babesia* spp.
		IFAT *n* (%)	CS-PCR *n* (%)	BC-PCR *n* (%)	IFAT *n* (%)	BC-PCR *n* (%)	IFAT *n* (%)	BC-PCR *n* (%)	BC-PCR *n* (%)	BC-PCR *n* (%)
Age										
< 1 year	32	0 (0%)^a^	0 (0%)^a^	0 (0)	0 (0)^a^	0 (0)	0 (0%)^a^	0 (0%)	0 (0)	0 (0)
≥ 1 year	254	31 (12.20%)^a^	45 (17.71%)^a^	0 (0)	18 (7.08)^a^	0 (0)	23 (9.05%)	0 (0%)	0 (0)	0 (0)
Sex										
Male	124	5 (4.03)	18 (14.51)	0 (0)	3 (2.41)	0 (0)	4 (3.22)	0 (0)	0 (0)	0 (0)
Female	162	26 (9.09)	27 (16.66)	0 (0)	15 (9.09)	0 (0)	19 (31.48)	0 (0)	0 (0)	0 (0)
Housing type										
Cattery	112	8 (7.14%)^a^	10 (8.92)^a^	0 (0)	12 (13.39)^a^	0 (0)	15^a^ (13.39)	0 (0)	0 (0)	0 (0)
Colony	174	23 (13.21%)^a^	35(20.11)^a^	0 (0)	6 (3.44)^a^	0 (0)	8 (4.59)^a^	0 (0)	0 (0)	0 (0)
Geographical origin										
Perugia (Umbria)	166	6 (3.61)^a^	7 (4.21)^a^	0 (0)	17 (10.24)	0 (0)	19 (11.44)	0 (0)	0 (0)	0 (0)
Macerata (Marche)	22	1 (4.54)^a^	0 (0)^a^	0 (0)	0 (0)	0 (0)	2 (9.09)	0 (0)	0 (0)	0 (0)
Arezzo (Tuscany)	98	24 (24.48)^a^	38 (38.77)^a^	0 (0)	1 (1.02)	0 (0)	2 (2.04)	0 (0)	0 (0)	0 (0)
Ectoparasitic status										
Infested	167	20 (11.97)	29 (17.36)	0 (0)	13 (7.78)	0 (0)	19 (11.38)^a^	0 (0)	0 (0)	0 (0)
Not infested	119	11 (9.24)	16 (13.45)	0 (0)	5 (4.2)	0 (0)	4 (3.36)^a^	0 (0)	0 (0)	0 (0)

^a^Significant differences by Pearsonʼs Chi-square test and Fisher’s exact test

*Abbreviations*: IFAT, immunofluorescence antibody assay; CS-PCR, conjunctival swab-PCR; BC-PCR, buffy coat-PCR

## Discussion

Feline-VBPs occurrence and distribution among Italian cat populations are described in many studies but with a focus on southern regions and insular areas [1, 2, 5, 12, 30, 32, 45]. Therefore, the present study adds new information on the occurrence of emerging FeVBPs in central Italy, an area that to date has received little attention.

The rates of antibodies and/or DNA of microorganisms demonstrate the exposure of cats to VBPs, some of which (e.g. *R. conorii*, *L. infantum*) have already been serologically and molecularly detected in canine populations from the same geographical areas [36, 37]. It is difficult to compare the infection prevalence between canine and feline species. In fact, the different study designs, and above all the different immune responses, host preferences of the vectors for feeding, or the innate resistance to VBPs might account for the differences in prevalence rates between cats and dogs sharing the same habitats.

The *Rickettsia conorii* serological rate (6.29%) detected in the present study is quite low, especially if compared with rates observed in Italian southern and insular regions (as high as 48.7%) [12]. However, the seroreactivity for *R. conorii* in cats is known has a wide variability, from 1.9 to 44% [7, 8, 12]. For instance, in seroprevalence surveys conducted in cats from Spain, a 44% positivity rate was detected, which was not significantly different from that recorded in dogs from the same geographical areas [46]. In accordance with these studies, the rate of seropositivity detected in our study is quite similar to that recorded in the domestic canine population (11%) living in the same geographical areas [36]. Some authors have found a significantly lower feline seroprevalence than that detected in dogs, speculating that cats are more resistant to rickettsioses than dogs [47]. However, it is still unknown whether cats are indeed less susceptible to infection or are infected less frequently because they remove ticks during self-grooming, or whether these infections are underreported in feline species.

In the present work, 23 sera (8%) tested positive for *R. felis* antibodies, which is in accordance with the seroprevalences previously observed in naturally-exposed cats in the USA (from 7.7 to 11.1%) [15, 48]. There are no data on canine exposure from the same geographical areas, because to date no epidemiological investigations on dog exposure to *R. felis* in Italy have been conducted.

The observed seroreactivities to rickettsiae had low antibody titers, reaching a maximum of 1/128, and five cats (1.74%) reacted to both pathogens with 1/64 (cut-off antibody titer).

The present serosurvey was performed using IFAT, a serological tool that is considered the gold-standard serological technique for rickettsioses in dogs, and is also commonly employed to assess the antibody response in cats. Since there is a considerable cross-reactivity between the numerous SFGRs in IFA tests [7] and no DNA was detected from feline blood by a molecular assay, it was not possible to identify with absolute certainty the infection source that stimulated the antibody response in the sampled cats.

The lack of concordance between serological and molecular tools is not unexpected. In fact, several studies have failed to detect SFGRs in the blood of dogs with suspected rickettsiosis [49] as they are only rickettsemic for short periods [50]. Little is known about the development and duration of rickettsiemia in dogs and even less so in cats. The fact that cats are mostly found to be PCR-negative in blood may be due to: (i) a rapid and effective immune response that neutralizes the pathogens; or (ii) that the organisms are sequestered in other tissues, for instance the spleen [48, 51–53].

With regard to the results obtained by the univariate analysis, age and living arrangements were significant factors associated with seropositivity for both *R. felis* and *R. conorii*. In fact, cats older than one year and cattery cats had a higher seroprevalence than animals younger than one year and colony cats, respectively. These animal-level factors may be correlated to the time spent in environments where fleas and ticks are prevalent, in particular in catteries where ectoparasites with endophylic behaviour, i.e. *R. sanguineus* and *C. felis*, are prevalent which thus increased the level of exposure of cats to vectors and tested pathogens.

*Cytauxzoon* spp. is the only FeVBP among those investigated that is not of public concern; however, we decided to investigate it because *Cytauxzoon* spp. DNA was previously detected in the spleen of two wild cats from the same geographical areas where the present survey was conducted [22]. Since a strong association was detected between high *Cytauxzoon* spp. PCR positivity rates (23–30%) in cat populations and the occurrence of infected wild felid reservoirs [23, 54], it was presumed that this piroplasmid agent was present in our study. Although no DNA was detected from feline BCs by molecular assay in the present survey, it is not possible to rule out that *Cytauxzoon* spp. may represent a risk among FeVBPs because a limitation of the results obtained consists on the fact that BC is not the optimal matrix for *Cytauxzoon* spp. detection. The BC in fact allows the detection of the mature schizonts within the monocytes but previous studies have shown that the schizogonous cycle of *Cytauxzoon* spp. is likely to be time-limited in relation to the intraerythrocytic cycle (corresponding to a prolonged erytroparasitemia) [55]. Nevertheless, large-scale investigations conducted on stray cats from the islands and northern regions of Italy have showed a 0% prevalence [12, 27] which is in-line with the present survey.

Previous studies have reported a variety of Babesia pathogens in wild and domestic cats around the world; however, in the present survey no serological and/or molecular evidence of *Babesia* spp. was observed. On the other hand, 20.8% antibody prevalence for *B. microti* has been detected in cats from Sicily [5]. The most prevalent FeVBP in terms of epidemiological aspects, veterinary and human health found in the present survey was *L. infantum*. In Mediterranean areas, cats are infected by the same *Leishmania* species as dogs; however, the FeL prevalence is lower than CanL, and cases of disease are less frequently reported [1, 30]. Little information is available on the mechanisms responsible for the susceptibility or resistance of feline hosts naturally exposed to *L. infantum* infection. This is probably due to the differences in the immune system of these two species and to a more efficient Th1 immune response in cats compared to dogs. Both serological and molecular investigations are commonly used with the same methodologies for both CanL and FeL; however, not much is known about their diagnostic performance in FeL [56]. The investigated areas were selected in CanL stable endemic foci [37, 39]. However, they included foci characterized by a different risk of endemicity from medium-low to high risk [57]. Surveys on FeL carried out in Mediterranean areas where CanL is endemic revealed a wide range of prevalence rates; this striking variation may be due to vector- and/or reservoir-related factors but may be also attributed to differences among serological tests performed and their cut-off values, as well as among the molecular targets and PCR protocols used [58]. Several studies conducted in Italy the evaluated antibody and or molecular prevalence of FeL, reporting a wide range positivity, 2–59% for serology and 2–61% for molecular tools [12, 45]. In the present study, an overall FeL prevalence of 21.67% was recorded. However, the performance of each assay was different, highlighting the limited diagnostic value of BC n-PCR in cats. In fact, the rate of *Leishmania* infection detected by CS n-PCR (15.73%) was higher compared with that recorded by IFAT (9.09%) and negative in BC n-PCR (Table 2). The negative DNA detection in BCs is not unexpected. In fact, most feline epidemiological investigations have shown an inconstant or lower positivity rate of peripheral blood PCR in cats compared to dogs [1, 12, 32, 59]. It is probable that the parasite load in peripheral blood is variable over the course of infection. FeL serological and molecular data confirm the higher risk of Tuscany region with IFAT 24.48% and CS-nPCR 38.77% compared to Umbria (3.61 and 4.21%, respectively) and Marche (4.54 and 0%, respectively) regions (Table 3), fitting well with CanL risk prevalence data [57]. When considering the CS-nPCR procedure, it is known as a very sensitive test for CanL diagnosis, being able to detect high positivity rates in both asymptomatic and sick dogs [37, 60–63] and shows a good relative performance and a high concordance in comparison to standard IFAT serology [37]. However, there are only limited data are available on FeL non-invasive sampling (conjunctival or oral swabs) [1, 12, 64, 65]. The overall CS n-PCR results obtained in the present study showed a moderate agreement (κ = 0.27) with serological findings, much lower than that found in dogs [37]. Also in other studies, when serological and molecular tests were used at the same time, discrepancies were found in cats as well as in dogs [8, 32, 66]. The moderate agreement between IFAT and CS-nPCR may depend on their different performances (sensitivity and specificity) which may be influenced by several factors. The presence of CS-nPCR positive but IFAT-negative cats, may indicate the presence of resistant animals and/or animals in the early stages of infection. On the other hand, IFAT-positive cats but negative by CS-nPCR may indicate that the animals had been previously exposed to *L.* *infantum* and developed specific IgG which was still detectable although the infection had cleared itself [67]. These findings suggest different values of the diagnostic markers in epidemiological survey and suggest to better investigate the suitable use of CS n-PCR in FeL clinical diagnosis or epidemiological studies as already reported in CanL surveys [37, 63]. The univariate analysis showed that cats older than one year have a significantly higher risk of being found positive to *L. infantum*. This may reflect the higher exposure of older animals due to continuous phlebotominae-bites in the further transmission seasons occurring in endemic areas. Cats from colonies showed a higher level of exposure to *L. infantum* than cats housed in catteries, and thus could still be correlated to the time spent in outdoor environments by the stray cats from colonies.

One limitation of the present study is the lack of investigation of the association between the presence of the FeVBPs and the occurrence of clinical signs. In fact, the good clinical conditions of the sampled feline population, representing criteria for the inclusion in the regional neutering projects, prevented this. The pathogenic effects of the FeVBPs of interest are still unknown or uncertain in cats, with the exception of *L. infantum*. However, several previous investigations have also failed to establish a clear association between the clinical status of cats (healthy or sick) or the presence of various clinical signs and the results of serology also for *L. infantum* [8, 67–70]. Only a few studies found that only skin lesions compatible with leishmaniosis were a clear risk factor for seropositivity [71].

## Conclusions

To our knowledge this is the first serological and molecular survey on the prevalence of such a wide range of FeVBPs in feline populations in central Italy. The results indicate the infective pressure of different FeVBPs within the studied areas and should alert owners, the veterinary community, and also public health authorities to the possible risk of potential zoonotic agents including *R. conorii*, *R. felis* and *L. infantum*. In the future, the priority should be to monitor cats from the studied areas to investigate the clinical and clinicopathological abnormalities associated with these FeVBPs and expand the spectrum of infectious agents to investigate.

## Abbreviations

FeVBP: feline vector-borne pathogen
CS: conjunctival swab
IFAT: indirect fluorescent antibody test
BC: buffy coat
n-PCR: nested-PCR
SFGR: spotted fever group rickettsiosis
VBP: vector-borne pathogen
FBSV: flea-borne spotted fever
ZVL: zoonotic visceral leishmaniasis
CanL: canine leishmaniosis
FeL: feline leishmaniosis
EDTA: ethylenediamine tetraacetic acid
IgG: immunoglobulin G
*SSU* rDNA: small subunit ribosomal DNA
κ: kappa coefficient
